# The Anti-Inflammatory and Anti-Oxidant Mechanisms of the Keap1/Nrf2/ARE Signaling Pathway in Chronic Diseases

**DOI:** 10.14336/AD.2018.0513

**Published:** 2019-06-01

**Authors:** Wenjun Tu, Hong Wang, Song Li, Qiang Liu, Hong Sha

**Affiliations:** ^1^Institute of Radiation Medicine, China Academy of Medical Science & Peking Union Medical College, Tianjin, China; ^2^Department of Neurosurgery, Beijing Tiantan Hospital of Capital Medical University, Beijing, China; ^3^Center for Translational Medicine, Institutes of Stroke, Weifang Medical University, Weifang, China; ^4^Institute of Biomedical Engineering, China Academy of Medical Science & Peking Union Medical College, Tianjin, China

**Keywords:** Oxidative stress, Reactive oxygen species, Keap1/Nrf2/ARE, Anti-inflammatory, Anti-oxidant, Low-level laser irradiation

## Abstract

Oxidative stress is defined as an imbalance between production of free radicals and reactive metabolites or [reactive oxygen species (ROS)] and their elimination by through protective mechanisms, including (antioxidants). This Such imbalance leads to damage of cells and important biomolecules and cells, with hence posing a potential adverse impact on the whole organism. At the center of the day-to-day biological response to oxidative stress is the Kelch-like ECH-associated protein 1 (Keap1) - nuclear factor erythroid 2-related factor 2 (Nrf2)- antioxidant response elements (ARE) pathway, which regulates the transcription of many several antioxidant genes that preserve cellular homeostasis and detoxification genes that process and eliminate carcinogens and toxins before they can cause damage. The redox-sensitive signaling system Keap1/Nrf2/ARE plays a key role in the maintenance of cellular homeostasis under stress, inflammatory, carcinogenic, and pro-apoptotic conditions, which allows us to consider it as a pharmacological target. Herein, we review and discuss the recent advancements in the regulation of the Keap1/Nrf2/ARE system, and its role under physiological and pathophysiological conditions, e.g. such as in exercise, diabetes, cardiovascular diseases, cancer, neurodegenerative disorders, stroke, liver and kidney system, etc. and such.

## 1. Oxidative stress

Oxidative stress, as a concept in redox biology and medicine, has been formulated in 1985 [1]. In the beginning of 2018, approximately 188 188,737 PubMed entries are show for displayed for this term. Oxidative stress is defined as an imbalance between the production of free radicals and reactive metabolites or [reactive oxygen species (ROS)] and their elimination by through protective mechanisms such as (antioxidants) [2]. This imbalance leads to damage of cells and important vital biomolecules and cells, with potential impact on the whole organism [3].

ROS, which are products of a normal cellular metabolism and, play vital roles in the stimulation of signaling pathways in plant and animal cells in response to changes in intra- and extra-cellular environmental conditions [4]. Most ROS are generated by the mitochondrial respiratory chain in cells [5]. During endogenous metabolic reactions, aerobic cells produce ROS, such as superoxide anion (O2-), hydrogen peroxide (H2O2), hydroxyl radical (OH•), and organic peroxides, as normal products of the biological reduction of molecular oxygen [6]. If ROS production increases beyond the threshold of this buffering capacity, these reactive species trigger uncontrolled reactions with non-target intracellular compounds, thus oxidizing nucleic acids, proteins, cellular membrane, and other lipids. Proteins and lipids are also significant targets for oxidative attack, and modification of these molecules can increase the risk of mutagenesis [7]. Increased levels of ROS cause oxidative stress and that severely damage lipids, proteins, and DNA [8].

Oxidative stress can activate a variety of transcription factors including nuclear factor kappa light chain enhancer of activated B cells (NF-κB), AP-1activator protein 1, p53, HIF-hypoxia-inducible factor 1α, peroxisome proliferator-activated receptor γ (PPAR-γ), β-catenin/Wnt, and Nrf2. Activation of these transcription factors can lead to the expression of over 500 different genes. Supraphysiologic levels of ROS that which exceed the capacity of the cellular radical-scavenging systems can cause oxidative stress and activate pro-inflammatory pathways [8]. ROS contribute to endothelial dysfunction and subsequent formation of atherosclerotic lesions [9]. Indeed, in vitro studies, as well as small in vivo trials, suggest that treatment with antioxidants chelators or antioxidative, enzymes might prevent ROS-mediated damage [8].

Previous studies had proposed that continuous oxidative stress can lead to chronic inflammation, which in turn could cause various chronic diseases. Increasing evidences in both experimental and clinical studies suggests that oxidative stress plays a major role in the pathogenesis of both types of diabetes mellitus [10]. Increased oxidative stress has been linked to impaired endothelial function in atherosclerosis and may play a role in the pathogenesis of cardiovascular events [11]. Endothelial dysfunction and increased vascular oxidative stress predict the risk of cardiovascular events in patients with coronary artery disease [11]. Endothelial dysfunction and increased vascular oxidative stress predict the risk of cardiovascular events in patients with coronary artery disease [12]. Furthermore, oxidative stress had been proposed to be involved in the pathogenesis of play role in many other diseases, such as cancer [7], neurological, and pulmonary diseases, age-related macular degeneration [13], systolic and diastolic heart failure [14], Alzheimer’s disease (AD) [15], Parkinson’s disease (PD) and amyotrophic lateral sclerosis [16], neurodegenerative diseases [17], microvascular and macrovascular complications in diabetic patients with diabetes [18], and cardiovascular events in patients with chronic kidney disease (CKD) [19].

**Figure 1.**
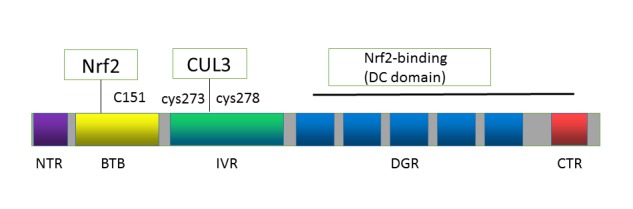
Domain structures of Keap1 Keap1 consists of three major functional domains: the BTB, IVR, and the Kelch/β-propeller domains.

## 2. Biology of Keap1/Nrf2/ARE pathway

At the center of the day-to-day biological response to oxidative stress is the Kelch-like ECH-associated protein 1 (Keap1) - nuclear factor erythroid 2-related factor 2 (Nrf2) - antioxidant response elements (ARE) pathway, which regulates the transcription of numerous antioxidant genes that preserve cellular homeostasis and detoxification genes that process and eliminate carcinogens and toxins before they can cause damage.

### 2.1 Keap1

Keap1 is a 624-amino acid, cysteine-rich, homodimeric zinc-finger protein that functions as an adapter for Cul3-Rbx E3 ubiquitin ligase complex (Fig. 1) [20]. Keap1 is composed by six domains, in which three are broad complex, tramtrack, bric-a-brac (BTB) domain, one is an intervening region (IVR), and two are glycine repeat domains (DGR) (Figure 1). Keap1 binds to the N-terminal Neh2 domain of Nrf2 through its Kelch domain, and the BTB domain of Keap1 recruits Cul3. The binding of Nrf2 to the DGR domain is competitively inhibited by proteins with specific motifs such as p62 and partner and localizer of BRCA2 [21]. The IVR domain, in addition to its interaction with Cul3 protein that contains the E3 ligase complex together with Roc1 [22], has a consensus sequence of nuclear export signal, which is important for localization of Keap1 at the cytoplasm [23]. Important cysteine residues for sensing electrophiles have been identified in the BTB (Cys151) and IVR (Cys273/288) domains [24].

**Figure 2.**
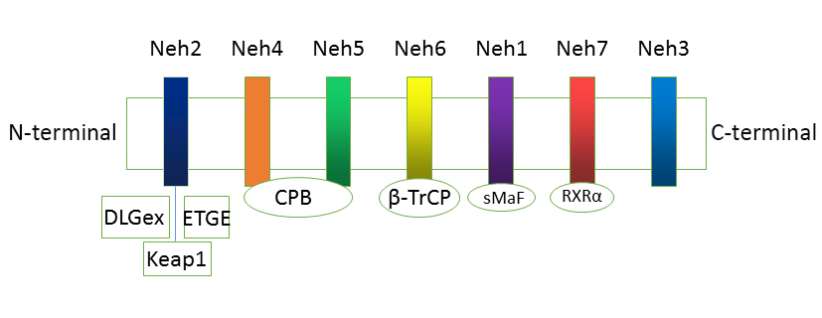
Domain structures of Nrf2 The Nrf2 protein contains7 domains, Neh1-Neh7. The ETGE and DLG motifs in the Neh2 domain are essential for the direct interaction with the Kelch domain of Keap1.

### 2.2 Nrf2

Nrf2 is a 605-amino acid transcription factor composed of seven functional domains (Neh1-7) (Fig. 2). The N-terminal Neh2 domain is the binding site for inhibitory protein Keap1, and it binds the Keap1 homodimer in two places [2]. It has a high affinity binding site with an ETGE motif and a low affinity site with a DLG motif separated by an alpha helix zone, which form the basis of the “latch and hinge” theory of Nrf2 activation [25-26]. The Neh1 domain, with its basic leucine zipper motif, allows the binding of Nrf2 to the ARE sequence [27-28]. The C-terminal of the Neh3 domain interacts with the transcription co-activator CHD6, which is a chromo-ATPase/helicase DNA-binding protein that is responsible for the transactivation of ARE-dependent genes after chromatin remodeling [29-31]. Neh4 and Neh5 represent domains of transcription activation that bind to the co-activator cyclic adenosine monophosphate (AMP)-responsive element-binding protein and facilitate Nrf2 transcription [31]. In addition, Neh4 and Neh5 can also interact with the nuclear cofactor RAC3/AIB1/SRC-3 and enhance Nrf2-targeted ARE gene expression [29-31]. Neh7 domain interacts with retinoic X receptor α, thus repressing Nrf2 [32].

### 2.3 Keap1/Nrf2/ARE pathway

**Figure 3.**
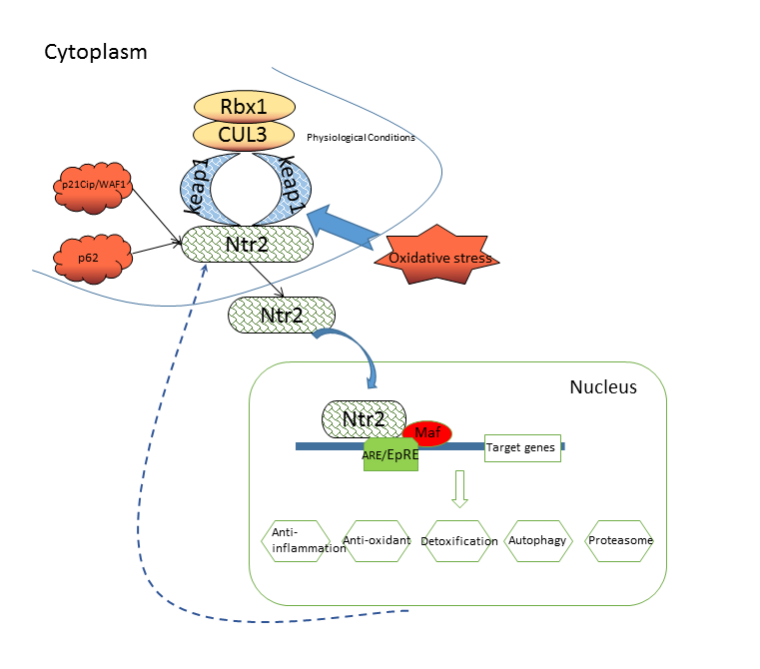
The Keap1-Nrf2-ARE pathway. Under physiological conditions, Nrf2 is restricted in the cytoplasm via its association with Keap1-Cul3-Rbx1 complex. In response to oxidative stress, Nrf2 is released from Keap1 translocates to the nucleus and heterodimerizes with one of the small Maf (musculoaponeurotic fibrosarcoma oncogene homolog) proteins. This complex activates the ARE-dependent gene expression of a series of antioxidative and cytoprotective proteins.

Nrf2 is a central player in the regulation of cellular defense mechanisms against environmental stresses [33]. Under normal physiological conditions, most Nrf2 is sequestered in the cytosol by its actin-bound inhibitor protein Keap1, a zinc metalloprotein that is localized near the plasma membrane [34-37]. Under oxidative stress, the Nrf2-Keap1 interaction is resolved in a dose-dependent manner, and the free and newly synthesized Nrf2 translocates to the nucleus and heterodimerizes with one of the small Maf (musculoaponeurotic fibrosarcoma oncogene homolog) proteins [38]. As shown in Figure 3, we propose that Nrf2/Maf complex activates the ARE-dependent gene expression of a series of antioxidative and cytoprotective proteins. Nrf2/Maf/ARE complex also play a physiological role through its anti-inflammatory, antioxidant, detoxification, autophagy, and proteasome actions.

Disruption of the interaction between Nrf2 and Keap1 is sufficient to trigger the activation of the Nrf2 pathway, as exemplified in the case of Nrf2 activation by the cyclin-dependent kinase inhibitor p21Cip/WAF1, which competes with Keap1 for binding to the DLG motif of Nrf2 and thereby protects Nrf2 from ubiquitination by opening the DLG latch [39]. p62 was identified as another protein that activates Nrf2 by disrupting the Keap1-Nrf2 interaction in autophagy-deficient cells (Fig. 3) [40]. Aside from Keap1, levels of active Nrf2 are regulated by autophagy and p62, which is a ubiquitin-binding protein acting as a scaffold for several protein aggregates and triggering their degradation through autophagy via the proteasome or the lysosome pathway [41]. p62 is degraded through autophagy under normal conditions. Oxidative stress upregulates p62 with resultant sequestration of Keap1 and activation of Nrf2 and Nrf2-dependent antioxidant defense gene expression [38]. In addition, protein kinase C, mitogen-activated protein kinases, and phosphotidylinositol 3-kinase (PI3K) have been implicated in the regulation of Nrf2/ARE signaling (Fig. 3) [42].

### 2.4 Downstream target protein regulated by Keap1/Nrf2/ARE signaling pathway

Nrf2 can evade Keap1-mediated degradation, translocate to the nucleus, and activate ARE-dependent gene expression of a series of antioxidative and cytoprotective proteins including heme oxygenase-1 (HO-1), NAD(P)H dehydrogenase, quinone 1 (NQO1), c-glutamylcysteine synthetase, glutathione peroxidase 1, glutathionine S-transferase (GST), glutathione reductase (GR), and superoxide dismutase (SOD) [43].

(1) Keap1/Nrf2/ARE regulates glutathione (GSH) levels by upregulating GSH synthetic and regenerative enzymes, including enzymes using GSH as a cofactor [2]. Glutamate cysteine ligase (GCL), including its two subunits GCLC and GCLM, catalyzes the rate-limiting step in GSH synthesis. Both GCLC and GCLM are upregulated by Nrf2 [44].

(2) SOD is a metalloenzyme (Zn, Cu-SOD) that is stabilized by zinc using copper as the redox agent in the active site. Manganese is the redox agent in mitochondrial SOD.

(3) NAD(P)H: NQO-1 is an inducible enzyme encoded by *NQO1* gene. Recent work suggests that the Nrf2-Keap1 pathway regulates cytosolic and mitochondrial ROS production. Nrf2 deficiency leads to enhanced NAPDPH oxidase 2 activity and unrestricted Nrf2 activation, as knocking down Keap1 leads to enhanced NADPH oxidase 4 activity [45], which highlights the essential role of Nrf2-Keap1 pair in redox homeostasis and that of NADPH oxidase in regulating Nrf2 [46-47].

(4) HO-1, encoded by the *HMOX1* gene, is an inducible enzyme that catalyzes the freeing of heme-bound Fe to form biliverdin. Biliverdin can then be reduced by biliverdin reductase to bilirubin, releasing carbon monoxide (CO) and exerting its anti-inflammatory effect. A previous study showed that the Nrf2-ARE pathway and its downstream antioxidant enzyme HO-1 are crucial for melanocytes to cope with H2O2-induced oxidative damage [48].

(5) Catalase is a highly efficient enzyme that reduces H2O2 to water and oxygen using Fe in the catalytic site [49-50].

(6) Thioredoxin (Trx) is a protein disulfide reductase that is itself reduced by thioredoxin reductase (TrxR) [51]. Nrf2 and the oxidoreductase thioredoxin-1 (Trx-1) have been previously identified as protective factors in cardiovascular disorders, with Trx-1 stimulating oxidative phosphorylation and tricarboxylic acid cycle via peroxisome proliferator-activated receptor gamma coactivator 1α and Nrf2 in cardiomyocytes and Nrf2 stimulating Trx-1 expression [52-53].

(7) In addition to direct upregulation of ARE-responsive genes, Nrf2 also supports antioxidant and detoxification pathways by increasing the synthesis and regeneration of NADPH, which is a niacin-derived reducing agent. NADPH is a direct antioxidant and is used as an enzyme cofactor in many redox reactions such as in GSH reduction by GR [54-55].

(8) Nrf2-deficient mice are more susceptible to benzo[α]pyrene-induced tumor formation, thus suggesting that Nrf2 is essential for a complete phase II metabolism [56]. Furthermore, the role of Nrf2 system in phase I-related genes and phase III xenobiotic transporters had also been proposed [57]. Nrf2 might play a role in the whole process of xenobiotic metabolism. Collectively, defending against xenobiotic metabolism and providing an efficient antioxidant system, Nrf2 can be considered as one of the main factors contributing to animal evolution in a changing environment [38]. On the other hand, the Nrf2/Keap1 system can be epigenetically regulated by DNA methylation, histone modification, and microRNAs, which add another layer of complexity to Nrf2 regulation and function [58].

(9) Nrf2 and NF-κB pathways regulate the physiological homeostasis of cellular redox status and responses to stress and inflammation [38]. Previous studies have suggested that Nrf2 plays a role in counteracting NF-κB-driven inflammatory response in many experimental models [59-61]. Rac1, which is activated by lipopolysaccharides, stimulates NF-κB to induce Nrf2, which in turn upregulates HO-1 expression. Then, HO-1 reduces the NF-κB inflammatory activity and shifts the cells to a more reducing environment that is essential for terminating the NF-κB activation [62-63].

## 3. Keap1/Nrf2/ARE pathway and disease implication

Keap1/Nrf2/ARE pathway represents one of the most important cellular defense mechanisms against oxidative stress and xenobiotic damage [64]. The Keap1/Nrf2/ARE pathway plays a major role in health resilience including inflammatory diseases [65], neurodegenerative diseases [66], PD [67], AD [68], stroke [69], chronic kidney disease [70], atherosclerosis [71], diabetes [72], cardiovascular diseases [73] and rheumatoid arthritis [74].

### 3.1 Exercise

By scavenging excessive ROS levels and restoring redox homeostasis, Nrf2 can prevent age-related muscular disorders and play a crucial role in response to training exercise [38]. In addition, uncontrolled Nrf2 activation can produce harmful consequences: In autophagic muscle disorders, Nrf2 is persistently activated with negative consequences on organ functions. Some studies had proposed that both resistance and endurance muscle exercises can lead to a perturbation of cellular redox homeostasis by increasing ROS and reactive nitrogen species [75-76]. Both *in vitro* (C2C12 skeletal muscle cells) and *in vivo* (rodent muscles) studies confirmed that oxidative stress can activate Nrf2 gene expression and transcriptional activity [75-79].

In young and older men, it has been shown that acute exercise can increase Nrf2 protein levels in peripheral blood mononuclear cells [80]. Moreover, nuclear accumulation of Nrf2 was observed only in the young group, thus indicating that aging is accompanied by a reduced nuclear import of Nrf2 [81-82]. Indeed, young animals showed no changes in Nrf2 expression, whereas older animals responded to the same training regimen with a decrease in Nrf2 [83]. Compared to young animals, older animals showed marked increases in baseline levels of Nrf2 expression [84]. Thus, it is plausible that resistance exercises, by restoring the redox homeostasis, lower Nrf2 basal levels. On the whole, exercise is an eligible routine to improve endogenous antioxidant defenses via Nrf2 activation. However, whether or not exogenous supplementation of antioxidants/Nrf2 activators during muscle adaptation is beneficial is still disputed [85-86]. Recent work has shown that the Nrf2 activator sulforaphane (SFN) enhances running capacity in rats by upregulating Nrf2 signaling and downstream genes and attenuates muscle fatigue via reduction of oxidative stress caused by exhaustive exercise [87]. Studies of Nrf2 activation as a response to resistance training produced less defined results, hence prompting further investigation.

Oxidative stress has long been implicated in the genesis of sarcopenia. ROS-activated NF-κB in myoblasts induces the expression of receptors for advanced glycation end-products (RAGE) that are required for timely muscle regeneration of acutely injured muscles, with the antioxidant N-acetyl cysteine reducing NF-κB activation and RAGE expression [88]. Moreover, Nrf2 transcriptionally upregulates carbonyl reductase 1, which plays a critical role in controlling redox balance and detoxifying lipid peroxidation during muscle differentiation and regeneration [89]. Lastly, mitochondrial ROS signaling repairs exercise-injured myofibers via RhoA-mediated F-actin assembly at injured sites [90]. Notably, Nrf2 activity was reported to reduce muscle glycogen content with resultant improved glucose tolerance via upregulation of the glycogen branching enzyme and muscle-type PhKα subunit mRNAs [38]. The protective effect of Nrf2 antioxidant pathway stimulation has been reported in other experimental settings such as in neuroinflammation [91].

### 3.2 Cardiovascular system

Oxidative stress plays a major role in the pathophysiology of cardiac disorders [92]. Several studies have highlighted the cardinal role played by ROS or RNS overproduction in the pathogenesis of ischemic myocardial damage and consequent cardiac dysfunction [93]. A previous study indicated that elevation of Tsg101 levels could be protective against myocardial ischemia/reperfusion (I/R) injury by activating the p62-Keap1-Nrf2 signaling [94]. A review summarized evidences from clinical studies and animal experiments relating to the potential mechanisms by which SFN modulates Nrf2 activation and protects against cardiovascular diseases [93]. Moreover, a series of studies reported that interventions against endoplasmic reticulum (ER) stress and Nrf2 activation reduce myocardial infarct size and cardiac hypertrophy in the transition to Heart failure (HF) in animals exposed to I/R injury and pressure overload, respectively [95]. Nonetheless, a negative side of Nrf2 had been proposed: when Nrf2 is over activated, it can cause and not prevent cardiovascular diseases [96]. Mounting evidence has strongly implicated oxidative stress in the development of cardiac dysfunction, and myocardial apoptosis contributes to the pathogenesis of heart failure [97]. ROS-mediated mitochondrial damage and cardiomyocyte apoptosis progress through modulations of Keap1-Nrf2 signaling axis [97]. A previous study demonstrated that butein and phloretin upregulate HO-1 and GCL expression through the ERK2/Nrf2 pathway and protect hepatocytes against oxidative stress [98]. Nrf2 activation in ischemia and I/R injury is being considered protective towards cardio-myocytes [38]. Nrf2 has been reported to operate downstream of NADPH oxidase-4, which is an important modulator of redox signaling that activates Nrf2-regulated pathway to regulate GSH redox in cardiomyocytes and to protect the heart in chronic hypertension [99-100]. Cardiac-specific Nrf2 knockout mice exhibited significantly dampened anti-apoptotic effects of tanshinone IIA sulfonate [97].

### 3.3 Diabetes and diabetic complications

In diabetes, oxidative stress impairs glucose uptake in muscles and fats and decreases insulin secretion from pancreatic cells [101-102]. Numerous evidences have recently elucidated the role of Keap1-Nrf2 system in metabolic and energy-balance regulation [103].

### 3.3.1 Oxidative stress damages pancreatic β-cells

Oxidative stress is enhanced in pancreatic islets of rodent models of diabetes, db/db mice, and Goto-Kakizaki rats [103]. A previous study reported that oxidative stress is increased in pancreatic islets of patients with diabetes [104]. Antioxidant reagents improve insulin secretion from pancreatic islets and ameliorate pancreatic b-cell damage in rodent diabetic models [105-106]. Thus, enhancement of antioxidant enzymes may be a useful strategy for pancreatic b-cell protection.

### 3.3.2 The Keap1-Nrf2 system in blood glucose homeostasis

The antioxidant function of the Keap1-Nrf2 system plays an important role in the maintenance of glucose metabolism through both insulin secretion and glucose utilization in insulin-sensitive tissues [103]. It has been reported that Nrf2 also regulates lipid metabolism-related genes [107]. Thus, Nrf2 plays an important role in maintaining glucose homeostasis in animal models [103]. Previous studies indicated that the Keap1-Nrf2 system did function in pancreatic b-cells and that Nrf2 regulates antioxidant enzyme gene transcription in pancreatic b-cells [103].

### 3.3.3 The Keap1-Nrf2 system protects pancreatic b-cells against stress through multiple critical pathways

First, the Nrf2-mediated antioxidant response is crucial for pancreatic b-cell protection against ROS/RNS and xenobiotics [108]. Second, the Nrf2-inducer SFN strongly suppresses cytokine-mediated iNOS and COX-2 induction, thereby ameliorating pancreatic b-cell damage [109]; thus, Nrf2 contributes to the suppression of inflammation in pancreatic b-cells. Third, Nrf2 also plays an important role in the maintenance of autophagy in pancreatic b-cells [110]. Fourth, Nrf2 regulates the expression of proteasome catalytic subunits and contributes to the ER stress response in pancreatic b-cells [111]. Finally, Nrf2 inducers enhance the phosphorylation of AMP-activated protein kinase (AMPK) and increase glucose uptake while suppressing glucose production in the liver, indicating that Nrf2 induction modulates AMPK signaling and improves insulin resistance [112]. In addition, previous findings suggest that activation of the Keap1-Nrf2 signaling pathway provokes anti-obesity effects, which may be an additional benefit in the clinical use of this pathway [103].

Several reports have claimed that Nrf2 depletion suppresses insulin resistance and obesity according to analysis of Nrf2 knockout mice. Blood glucose levels of Nrf2 knockout mice were lower than those of wild-type mice, and insulin signaling is enhanced in Nrf2 knockout mouse liver and skeletal muscle [103, 113]. However, some other studies found that Nrf2 depletion increased blood glucose levels [114]. Thus, multiple approaches are essential to elucidate the full array of Nrf2 functions.

### 3.3.4 Diabetic complications and the Keap1-Nrf2 system

Nrf2 depletion increases renal oxidative and nitrosative stress in streptozotocin (STZ)-induced mouse diabetes model [115]. Nrf2 also helps to prevent diabetic retinopathy and cardiomyopathy. In the STZ-induced diabetic model, the Keap1-Nrf2 system helped to protect against the onset and/or progression of diabetic retinopathy [116]. In addition, the Nrf2 inducer dihydro-CDDO-TFEA suppressed diabetes-mediated cardiac nitrosative damage [117].

### 3.4 Cancer

Comprehensive genomic analyses have found somatic mutations and other alterations in the *KEAP1* or *NRF2* genes and in well-known tumor suppressor genes or oncogenes, such as *TP53*, *CDKN2A*, *PTEN*, and *PIK3CA*, in various types of cancer [118]. Several studies have shown that Nrf2-Keap1 pathway protects against oxidative stress [119], chemotherapeutic agents [120], and radiotherapy [121] in cancer. However, Nrf2 disruption can also lead to the progression of inflammation and, ultimately, cancer formation [122]. Jeong et al. [123] found that *KEAP1*/*Nrf2* mutations increase radio resistance and predict local tumor recurrence in patients undergoing radiotherapy. Thus, this phenomenon is known as a ‘double-edged sword’, with respect to the benefits or risks of the Keap1-Nrf2 pathway in cells [124]. The discovery of the dual role of Nrf2-Keap1 pathway enabled scientists to understand Nrf2 signaling in cancer and to develop pharmacological compounds targeting Nrf2 for the prevention and treatment of cancer [122].

Elevated levels of Nrf2 in cancer cells can lead to: (1) Somatic mutations: gain-of-function mutations in Nrf2 and loss-of-function mutations in Keap1 and CUL3 have been identified in several human malignancies [122]. Somatic mutations in CUL3 were identified in cases of hereditary type 2 papillary renal cell carcinoma [125]; (2) Epigenetic silencing of Keap1 by hypermethylation: Methylation in the promoter region of Keap1 alters its expression and prevents its ability to bind with Nrf2. Conversely, DNA methylation by DNA methyl-transferases appears to indirectly downregulate Nrf2 expression [126-127]; (3) Accumulation of p21 and p62 disrupts the Nrf2-Keap1 complex: p53 negatively regulates Nrf2 [128]. p21, which is a direct downstream target of p53, associates with the DLG motif of Nrf2, leading to the disruption of Keap1 binding with Nrf2. Furthermore, p62 directly interacts with the kelch domain of Keap1 by its STGE motif that is similar to the Nrf2 ETGE motif, thereby disrupting the Keap1-Nrf2 complex [122]; (4) Transcriptional upregulation of Nrf2 by oncogenes: Oncogenes like *KRAS*, *BRAF*, and *C-MYC* increase mRNA levels of Nrf2 and its target genes [129]; (5) Metabolic activation of Nrf2 by Kreb cycle intermediates: Kreb’s cycle leads to prolonged activation of Nrf2 [130].

### 3.5 Neurodegenerative disorders

The protective effect of Nrf2 against neurodegeneration caused by oxidative stress has been well proposed. Studies on neurodegenerative diseases have found that activation of Nrf2 protects against H2O2-induced PC12 cell death and apoptosis [131-132].

### 3.5.1 Alzheimer’s disease

AD is a progressive neurodegenerative disease characterized by insidious cognitive decline and memory dysfunction. Studies have shown that the expression of Nrf2 target genes were increased in AD patients [122]. One study provided a strong evidence that direct Keap1-Nrf2 disruptors can specifically target the defects in Nrf2 activity observed in AD [133]. Another study showed a clear link between Nrf2 and AD-mediated cognitive decline, thus further strengthening the connection between Nrf2 and AD [134]. Furthermore, Lipton et al. [135] concluded that carnosic acid exhibits therapeutic benefits in rodent AD models by stimulating the Keap1/Nrf2 transcriptional pathway. The neuroprotective role of Nrf2 in AD had been mainly proposed through GSK-3β in the regulation of the Nrf2 pathway [136]. Therefore, the activation of Nrf2 could be a therapeutic target for AD and could potentially ameliorate this disease.

### 3.5.2 Parkinson’s disease

PD is a type of movement disorder, and it occurs when neurons in the brain do not produce enough dopamine. Activation of NOQ1 and HO-1, through Nrf2 nuclear localization, was induced in the substantia nigra of PD patients [137]. One study suggested that safranal protects against rotenone-induced neurotoxicity associated with the Nrf2 signaling pathway. This implies that safranal may be a potential therapeutic drug for the treatment of PD [138]. A link has been revealed between the transcription factor NRF2 and PD at genetic level; and it was shown that a functional haplotype in the human NFE2L2 gene promoter of NRF2, with slightly increased transcriptional activity, is associated with decreased risk and delayed age of onset of PD [139]. However, another study reported that NRF2 mRNA expression levels did not correlate with the rs35652124 genotype, PD, or age of onset in our material [140]. Furthermore, Lastres-Becker et al. [141] provided a compelling rationale for targeting NRF2 with dimethyl fumarate as a therapeutic strategy to reinforce endogenous brain defense mechanisms against PD-associated synucleinopathy.

### 3.6 Stroke

ROS has been widely reported to play a key role in the pathological process of ischemic stroke followed by reperfusion [142]. The role of Nrf2 in ischemic and hemorrhagic stroke had been proposed [143]. A previous study showed that recombinant human erythropoietin activates Keap1-Nrf2/ARE pathway after ischemia to protect the brain tissue [144]. Another study found that treatment with myricetin attenuates brain injury and neurological deficits in a rat model with cerebral ischemia *by* improving mitochondrial function and activating Nrf2 pathway [145]. Similarly, it was observed that in a mice model, hybrids (HP)-1c, with dual AMPK- and Nrf2-activating properties, may be potential subjects of further studies as novel therapy for ischemic stroke [146]. Loss of Nrf2 function increases the size of cerebral infarct and neurological deficits after an ischemic event [142]. Accumulating data suggest that the loss of Nrf2 exacerbates cerebral infarction and neurologic deficits in both middle cerebral artery occlusion (MCAO) and permanent MCAO models [147-150]. In addition, Nrf2 knockdown in endothelial cells significantly aggravated delocalization of the tight junction-associated protein ZO-1 during ischemic conditions, hence indicating the protective role of Nrf2 in blood-brain barrier integrity [151]. Thus, Nrf2 activation may protect neurons, astrocytes, oligodendrocytes, and microglia against oxidative stress; thus, the Keap1-Nrf2 pathway is suggested as one of the therapeutic approaches to the neurovascular system [122].

### 3.7 Liver and kidney system diseases

A previous study found that Nrf2 activation prevents alcohol-induced oxidative stress and accumulation of free fatty acids in liver by increasing genes involved in antioxidant defense and by decreasing genes involved in lipogenesis [152]. Another study confirmed that pretreatment with SFN could attenuate hepatic I/R injury via the activation of Nrf2/ARE signaling pathways, ameliorate oxidative stress, and maintain the normal activities of Na+-K+-ATPase and Ca2+-ATPase, thus reducing the occurrence of cell oncogenesis and apoptosis [153]. Shen et al. indicated that p62-Keap1-Nrf2 antioxidant pathway was primarily activated in the early stage of acetaminophen hepatotoxicity, which might play a protective role in the process of acetaminophen-induced acute liver injury [154]. In addition, therapeutic approaches to prevent oxidative stress via activation of the Nrf2-Keap1 signaling and/or suppression of uremic toxin-induced ROS production could be effective strategies for maintaining optimal kidney function [19].

### 3.8 Low-level laser irradiation, Oxidative stress and Keap1/Nrf2/ARE pathway

Low-level laser irradiation (LLLI) has gained increasing popularity as a treatment for soft tissue injuries and joint conditions [155]. It is applied transcutaneously with typical irradiances of 10-5,000 mW/cm2, treatments times ranging from 10 seconds to 2 minutes, and with total energy of 1-4 Joules(J)/cm2 per point delivered when targeting joints, tendons, and muscles [156]. Its application includes wound healing, healing of soft and hard tissues [157], and treating pain syndromes, enthesopathy, peripheral nerve injury, traumatic brain injury [158], and peripheral neuropathy, thus improving muscular performance [159]. LLLI modulates many biological processes, manifesting as an increase in mitochondrial respiration and in ATP synthesis proliferation of mesenchymal and cardiac stem cells [160]. The anti-inflammatory qualities of LLLI had also been suggested [161]. However, the biological effect of LLLI is still not well understood and is still controversial [162].

LLLI might play a role in the mitochondria, leading to increased ATP production, ROS modulation, and transcription factors induction. These effects, in turn, lead to increased cell proliferation and migration, particularly by fibroblasts [163]. Furthermore, previous studies in animal and human cell lines had proposed that LLLI play a role in anti-inflammatory and anti-oxidative stress responses [164-172]. (1) At the cellular level: Macedo et al. [164] suggested that the laser treatment improved regenerative capacity and decreased inflammatory response and oxidative stress in dystrophic muscle cells. Furthermore, Lubart et al. [165] demonstrated that various ROS and antioxidants were produced following low-energy visible light illumination. Similarly, Souza et al. [166] reported that LLLI acts by decreasing cytokines and histone deacetylase through the activation of protein kinase A via inhibition of PI3K in U937 cells. (2) In animal models: In both nondiabetic and diabetic rat models, LLLI was observed to possibly play pivotal roles in promoting wound healing process by improving glycemic state, cytokines involved in inflammation, and antioxidant defense system [167]. Another study indicated that LLLI therapy was efficient in accelerating skin wound healing process after wounding, and this is probably due to reduction of the inflammatory phase and induction of collagen synthesis in an animal model [168]. Furthermore, LLLI could be an effective therapeutic approach in modulating oxidative and nitrative stress and in reducing inflammation in rats with injured muscles [169]. Another study found that the effect of laser in attenuating acute lung inflammation restores the balance between the pro- and antioxidants mediators such as PPARy expression and consequently heat shock protein 70 production in rats [170]. (3) In human models: De Marchi et al. [171] found that the use of LLLI before progressive-intensity running exercise increases exercise performance and decreases exercise-induced oxidative stress and muscle damage in humans. Another study reported that LLLI reduced oxidative stress in neural and muscular tissues of non-obese volunteers [172].

Given the role of Keap1-Nrf2-ARE pathway and its anti-inflammatory and anti-oxidant effects, we hypothesize that the anti-inflammatory and anti-oxidant mechanisms of LLLI may be achieved by activating the Keap1/Nrf2/ARE signaling pathway. Hence, this would be the focus of our future research.

## 4. Conclusions

In this study, we review and discuss recent advancements in the regulation of the Keap1/Nrf2/ARE system and its role under physiological and pathophysiological conditions such as in exercise, diabetes, cardiovascular diseases, cancer, neurodegenerative disorders, stroke, liver and kidney system, etc. The redox-sensitive signaling system Keap1/Nrf2/ARE plays a key role in maintenance of cellular homeostasis under stress and in inflammatory, carcinogenic, and pro-apoptotic conditions, thus allowing us to consider this system as a pharmacological target. Furthermore, we also suggest LLLI as a potential therapeutic target to oxidative stress by regulating the Keap1/Nrf2/ARE system.
